# Intraindividual evaluation of effects of image filter function on image quality in coronary computed tomography angiography

**DOI:** 10.3389/fcvm.2022.840735

**Published:** 2022-09-15

**Authors:** Liang Jin, Pan Gao, Kun Wang, Jianying Li, Ming Li

**Affiliations:** ^1^Radiology Department, Huadong Hospital, Affiliated to Fudan University, Shanghai, China; ^2^CT Research Center, GE Healthcare China, Beijing, China; ^3^Institute of Functional and Molecular Medical Imaging, Fudan University, Shanghai, China

**Keywords:** coronary artery disease, X-ray computed tomography, analysis, sharpness, image quality

## Abstract

**Objectives:**

To evaluate whether applying image filters (smooth 3D+ and edge-2) improves image quality in coronary CT angiography (CCTA).

**Methods:**

Ninety patients (routine group) with suspected coronary artery diseases based on 16-cm wide coverage detector CT findings were retrospectively enrolled at a chest pain center from December 2019 to September 2021. Two image filters, smooth 3D+ and edge-2 available on the Advantage Workstation (AW) were subsequently applied to the images to generate the research group (SE group). Quantitative parameters, including CT value, signal-to-noise ratio (SNR) and contrast-to-noise ratio (CNR), image sharpness and image quality score, and diagnostic accuracy were compared between the two groups.

**Results:**

A total of 900 segments from 270 coronary arteries in 90 patients were analyzed. SNR, CNR, and image sharpness for vessels and image quality scores in the SE group were significantly better than those in the routine group (all *p* < 0.001). The SE group showed a slightly higher negative predictive value (NPV) on the left anterior descending artery and right coronary artery (RCA) stenosis evaluations, as well as total NPV. The SE group also showed slightly higher sensitivity and accuracy than the routine group on RCA stenosis evaluation.

**Conclusion:**

The use of an image filter combining smooth 3D+ and edge-2 on an AW could improve the image quality of CCTA and increase radiologists' diagnostic confidence.

## Introduction

Coronary computed tomography angiography (CCTA) has been widely used for detecting coronary artery stenosis in patients with suspicious coronary artery disease (CAD) (1–3). However, motion artifacts and image noise decrease CCTA image quality (4–7), and thus affect the diagnosis, management, and treatment of CAD (8). Notably, 16-cm-wide coverage-detector CT with motion-corrected reconstruction algorithm Snapshot Freeze (SSF) has been shown to reduce respiratory movement and motion artifacts (9–16). However, the image noise due to insufficient photon quantity in low-dose CCTA still needs to be overcome (17). Common ways to reduce image noise without increasing radiation dose include the use of iterative reconstruction algorithms (IRs), deep learning-based image reconstruction algorithms, and low-pass reconstruction kernels (RKs) in CCTA (18).

On the other hand, image filters (19) operated in the image domain also provide several options that could be applied through the post-processing procedure to reduce image noises and enhance edges (edge sharpening). Image sharpness as a quantitative index is important to evaluate the image quality (20–23). However, the use of image filters has not been focused on and whether this application of image filters could improve image quality and diagnostic accuracy remains unknown and was not reported before. Therefore, we hypothesized the improved diagnostic image quality through image filters, and the purpose of this study was to compare quantitative measurements, qualitative image score, diagnostic accuracy through CAD-RADS report guideline (24), and image sharpness between CCTA images with the combination of image smoothing and edge-enhancement filters and those without.

## Materials and methods

### Patients

**Figure 1:**
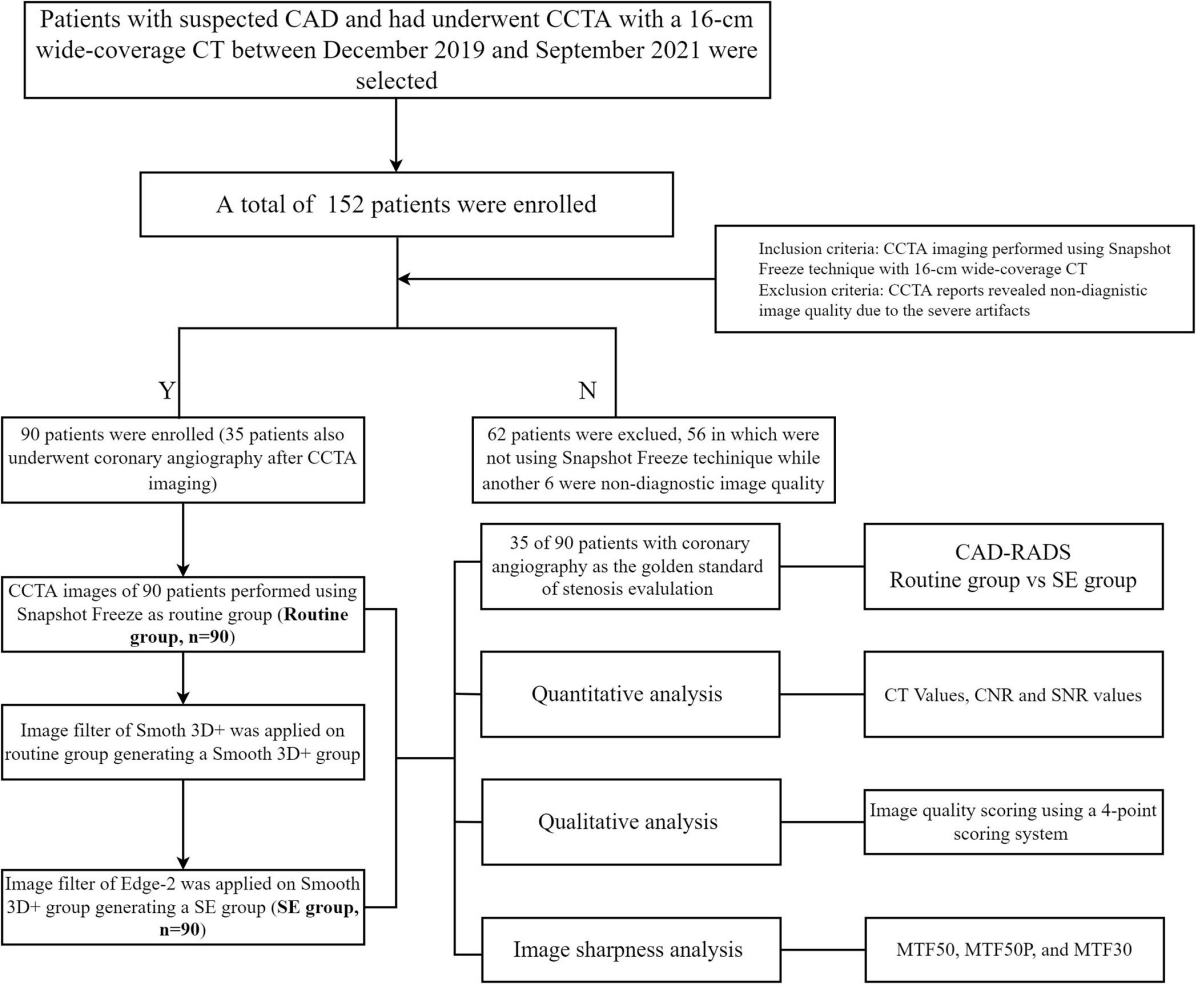
The flow chart of the patients' enrollment.

This single-center, retrospective study was approved by the ethics committee of our hospital, and the requirement for written informed consent was waived owing to the retrospective nature of the study. Patients with suspected coronary heart disease due to chest pain who underwent CCTA at our center between December 2019 and September 2021 were consecutively selected from the hospital radiology information system. Inclusion criteria included CCTA imaging performed using the SSF technique with CT (GE Healthcare). Exclusion criteria included the image that does not meet the diagnostic requirements due to motion artifacts. A total of 90 patients (23 women; mean age: 67.1 ± 11.7 [range 38–90] years) were enrolled for image quality evaluation and 35 of the 90 patients who undergone digital subtraction angiography (DSA) were also used for diagnostic accuracy evaluation (Figure 1). Of these 35 patients, 28 patients' main lesion caused significant coronary stenosis is mixed plaque and 7 patients' is non-calcified plaque.

### Image acquisition and reconstruction

All patients underwent wide-coverage-detector CT (Revolution CT, GE Healthcare) with the following parameters: tube voltage, 100–120 kV; automatic tube current modulation, 300–800 mA; data acquisition window (R–R interval), 30–80%; and detector coverage, 100 mm, 120 mm, 140 mm, or 160 mm dependent on patient heart sizes. The gantry rotation speed was 0.28 s/rotation.

**Figure 2:**
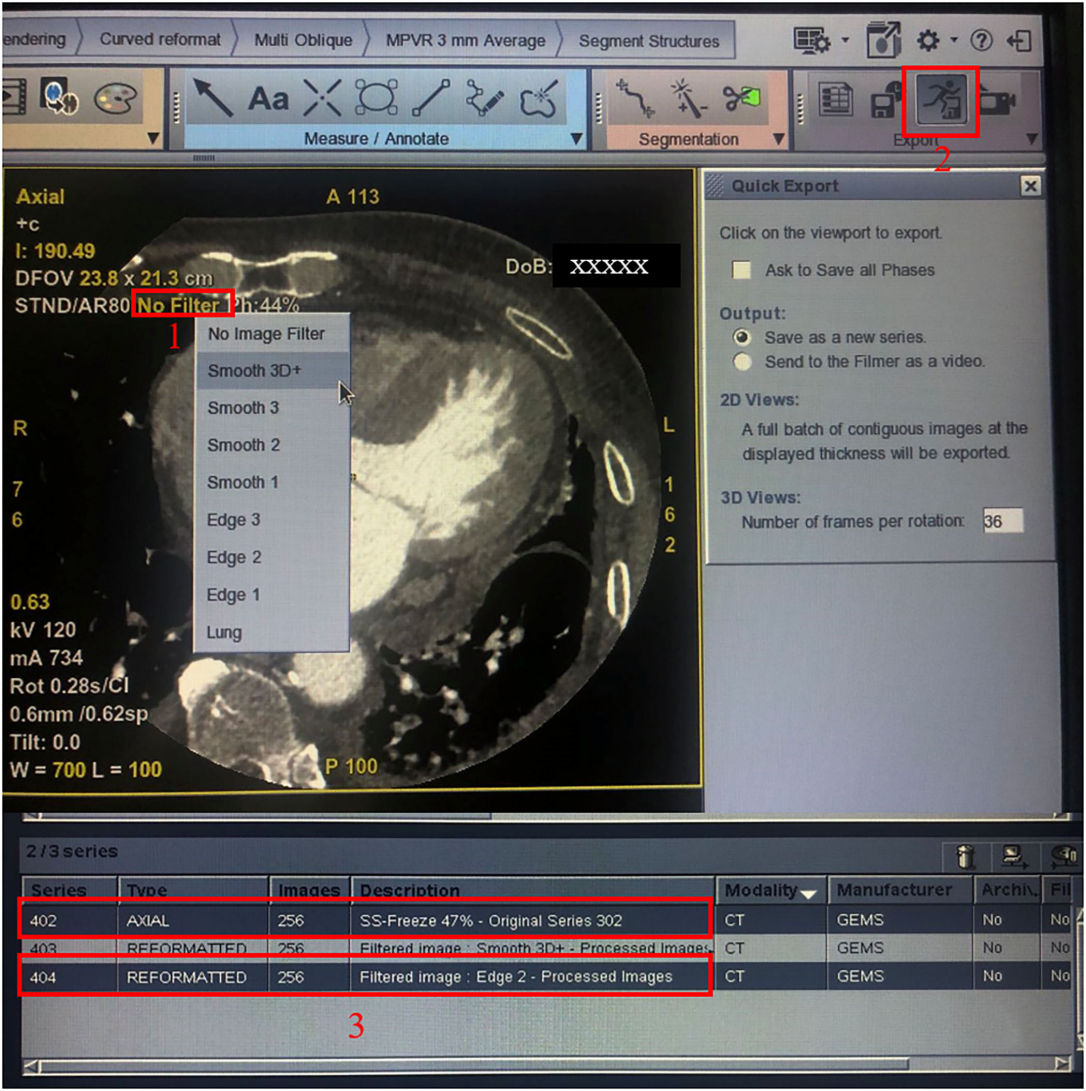
Procedure for generating the SE group. Step 1: Click the “No Filter” option in red frame 1 and select smooth 3D+; step 2: Click the button in red frame 2 to generate the smoothed 3D+ group. Then, quit the software program and start again, repeat step 1 but choose the edge-2 filter, and repeat step 2 and generate the SE group. Finally, the original series 402 and the SE group 404 were showed in two red frame 3.

CCTA images with a slice thickness of 0.625 mm and increment of 0.625 mm were reconstructed using the 80% adaptive statistical iterative reconstruction-V [ASIR-V80%, GE Healthcare] with the STND kernel at the optimal reconstruction phase automatically selected by the SmartPhase technique (GE Healthcare), and the cardiac motion correction technique (snapshot freeze, SSF) was also applied (12, 15, 17). The generated images were assigned as the routine group. Subsequently, all the CCTA images of the routine group were sent to an Advantage Workstation (AW4.7, GE Healthcare) to undergo further image filtering. After our preliminary exploration and comparison with different combinations of smooth and edge enhancement filters that are readily available on the AW4.7, the filter combination was narrowed down to the combination of smooth 3D+ and edge-2. On the workstation, the volume rendering software was selected, and the Smooth 3D+ image filter option was selected for noise reduction. The resulting images were subsequently saved as a new group named Smooth 3D+. In a second pass, volume rendering software was once again selected, and the Edge-2 image filter option was chosen for edge sharpening of the Smooth 3D+ group. The resulting images were saved as a new group named the smooth 3D+ and edge-2 group (SE group). The signal-to-noise ratio (SNR), contrast-to-noise ratio (CNR), and image quality scores were then compared between the original routine group and the SE group (Figure 2).

### Quantitative analysis

Quantitative measurements were performed including CT values and standard deviation (SD) of the lumen of the aortic root (AO), proximal left anterior descending artery (LAD-P), middle left anterior descending artery (LAD-M), distal left anterior descending artery (LAD-D), proximal left circumflex artery (LCX-P), middle left circumflex artery (LCX-M), distal left circumflex artery (LCX-D), proximal right coronary artery (RCA-P), middle right coronary artery (RCA-M), distal-proximal right coronary artery (RCA-D), and perivascular adipose tissue (PVAT). The region of interest (ROI) in the AO was set to 90 mm^2^, and those in others were set to 1 mm^2^. Image noise was classified as the SD of the attenuation within the ROI in the AO. Each ROI was measured three times by an independent reader, and the average of the three measurements was used for further analysis. The SNR was defined as CT value (vessel)/image noise, and the CNR was defined as [CT value (vessel) - CT value (PVAT)]/SD(PVAT).

### Qualitative analysis

Double-blinded image quality scoring was performed by two radiologists, one with 8 years of experience and the other with more than 15 years of experience in cardiovascular diagnosis, using the 18-segment model of the Society of Cardiovascular Computed Tomography guidelines (25). Evaluations were conducted over 1 month to eliminate the observers' recall effects between routine and SE groups of the same patient. A 4-point scoring system was used for overall image quality analysis (26, 27) [(1) excellent, no artifacts; (2) good, minor artifacts; (3) moderate, artifacts, but diagnosis still possible; and 4: poor, non-diagnostic]. Each patient's CCTA was evaluated through the following 10 segments for the severity of stenosis: left main artery, LAD-P, LAD-M, LAD-D, LCX-P, LCX-D, RCA-P, RCA-M, RCA-D, and posterior descending artery. The stenosis degree was assessed using Coronary Artery Disease - Reporting and Data System (CAD-RADS) with a 7-grade scoring system (0–2: non-significant stenosis [luminal irregularities or lumen diameter narrowing <50%]; 3–5: significant stenosis [lumen diameter narrowing ≥50%]; 6: non-diagnostic situation) (Table 1). In cases of disagreement on the image quality score or stenosis assessment between the two radiologists, a third experienced observer (more than 15 years of experience) would mediate to reach a final agreement. Individual adjustments of the window center and window width were allowed.

**Table 1:** Diagnostic criteria for coronary stenosis by CCTA and DSA.

	**Degree of maximal coronary stenosis**	**Interpretation**
CAD-RADS 0	0% (No plaque or stenosis)	Documented absence of CAD
CAD-RADS 1	1–24% - Minimal stenosis or plaque	Minimal non-obstructive CAD
CAD-RADS 2	25–49% - Mild stenosis	Mild non-obstructive CAD
CAD-RADS 3	50–69% stenosis	Moderate stenosis
CAD-RADS 4	70–99% stenosis	Severe stenosis
CAD-RADS 5	100% (total occlusion)	Total coronary occlusion
CAD-RADS 6	Non-diagnostic study	Obstructive CAD cannot be excluded

CAD-RADS, Coronary Artery Disease - Reporting and Data System; CCTA, Coronary computed tomography angiography; DSA, digital subtraction angiography.

The results of digital subtraction angiography (DSA) were used as the reference standard to determine the significant stenosis independent of the CCTA results. Patients underwent DSA for diagnostic accuracy by two cardiologists (with more than 10 years of experience). The stenosis degree of each segment of coronary vessels (range: 0–100%) was quantitatively evaluated. Similar to CCTA, all segments were classified as being non-significant stenosis, significant stenosis, or non-diagnostic situation.

### Image sharpness analysis

**Figure 3:**
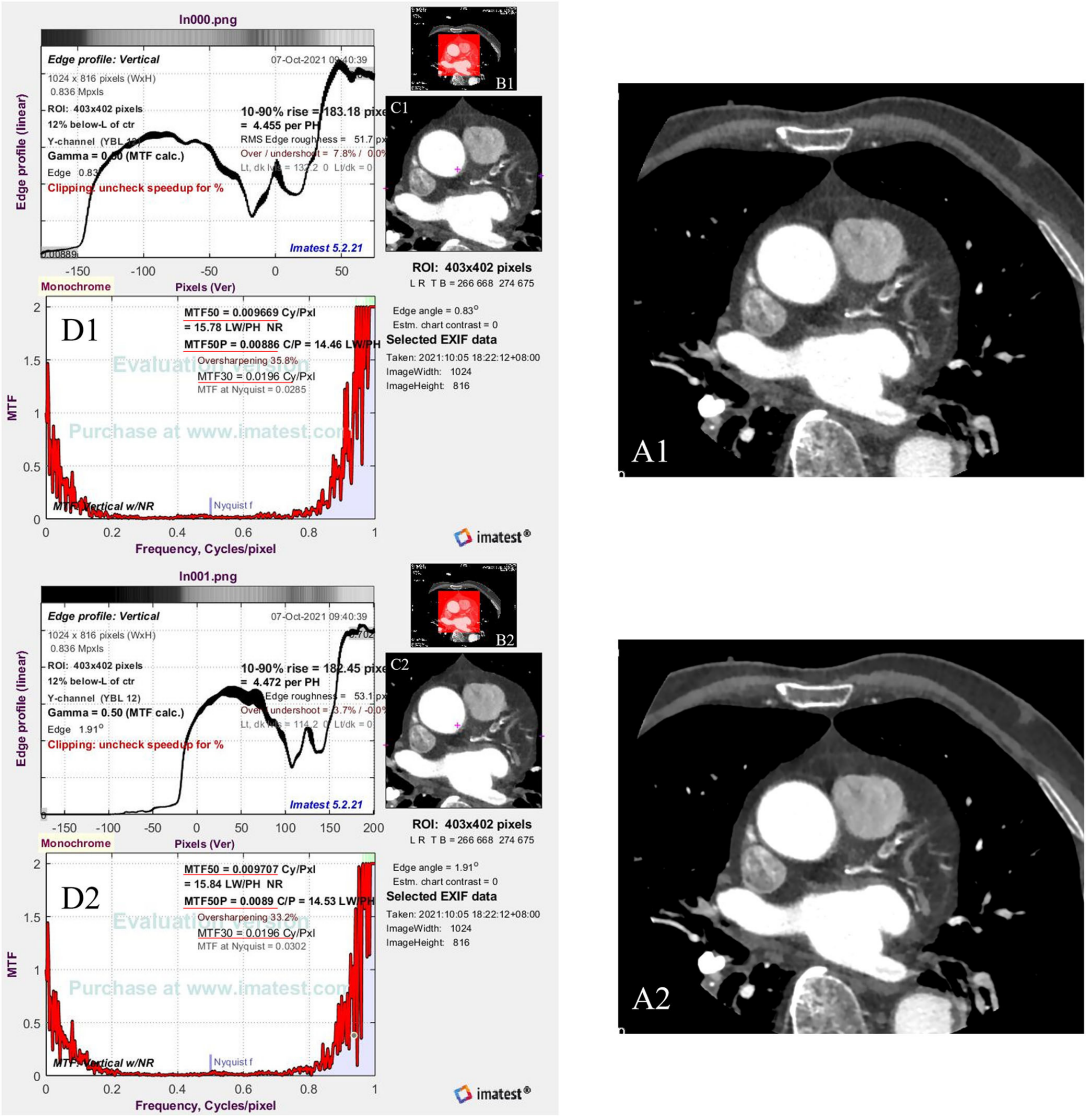
The flow chart shows how to calculate the image sharpness. **(A1,A2)** are the same Digital Imaging and Communications in Medicine (DICOM) slices of the beginning of the coronary sinus of one patient [**(A1)** from the routine group and **(A2)** from the SE group], which are exported to be a PNG format (Portable Network Graphic). Red frames in **(B1,B2)** are the setting of ROI (region of interest) **(C1,C2)**. Then, the software calculates the image sharpness through the ROI based on the MTF50, MTF50P, and MTF 30 [red underlines in **(D1,D2)**].

An image quality testing software (Imatest, V5.2.21, USA) was used to evaluate the spatial frequency response reflecting the image sharpness through modulation transfer function (MTF) in the 2D image domain (28). One radiologist chose the same Digital Imaging and Communications in Medicine (DICOM) slices of the beginning of the coronary sinus of every patient in each group (one from the routine group and one from the SE group) and then exported this image as a 2D image (Portable Network Graphic, PNG) for further image sharpness evaluation. Using the Imatest software, the same ROI was set on the same position of two 2D images, including the heart, and the MTF50, MTF50P, and MTF30 were then calculated automatically by the software (Figure 3).

### Radiation dose

The radiation dose of CCTA imaging, including the dose-length product (DLP) and the volume CT dose index was recorded through the dose reports provided by the CT scanner. The effective dose was estimated by multiplying the DLP by a conversion factor of 0.014 mSv/(mGy·cm) (29).

### Statistical analysis

SPSS Statistics V22.0 (IBM, Chicago, IL, USA) was used for statistical analysis. Quantitative indices are presented as mean ± SD and medians (range: maximum to minimum). Data with non-normal distribution are expressed as medians (interquartile ranges). Quantitative data were analyzed using the independent samples t-test or the Wilcoxon signed rank-sum test. Counts were assessed using Pearson's chi-squared test, and Fisher's exact test was employed to examine the probability when the expected value was <5. A kappa value was calculated and defined as follows: < 0.20, almost inconsistent; 0.21–0.40, slightly consistent; 0.41–0.60, medium consistency; 0.61–0.80, good consistency; and 0.81–1.00, perfect consistency (27). The false-positive rate, false-negative rate, positive predictive value, negative predictive value (NPV), sensitivity, specificity, and accuracy were calculated for CCTA vs. DSA for each reconstruction on per-vessel and total levels and analyzed by the χ^2^ test, and differences were analyzed with ANOVA between three groups. The significance level was set at *p* < 0.05.

## Results

In total, we analyzed 90 patients, 900 vessel segments, and 270 images of coronary arteries with scoreable quality. The mean DLP was 345.18 ± 94.74 mGy·cm, and the mean effective dose was 4.83 ± 1.33 mSv. Patients' characteristics are presented in Table 2.

**Table 2:** Patients' information and radiation dose.

Age	67.1 ± 11.7
Sex (F/M)	23/67
BMI (kg/m^2^)	27.4 ± 4.9
Smoker	56 (62%)
Hypertension	70 (78%)
Diabetes	61 (68)
Hyperlipemia	39 (43%)
DLP, (mGy)	344.69 ± 103.07
ED, (mSv)	4.8 ± 1.4

F/M, Female/Male; BMI, Body Mass Index; DLP, Dose-length Product; ED, Effective radiation dose.

### Quantitative analysis, image quality scores, and image sharpness

The CT value of the AO ROI and the image noise in the routine group were higher than those in the SE group (*P* < 0.001). Moreover, the SNR and CNR were significantly higher in the SE group than in the routine group (all *Ps* < 0.05). The SE group had significantly higher MTF50 and MTF50P values than the routine group, while the MTF30 values were similar in these two groups (Table 3).

**Table 3:** Comparison of sharpness of image between the routine and SE groups.

**CT values**	**Routine group (*n* = 90)**	**SE group (*n* = 90)**	***p-*value**
AO (HU)	409.55 ± 77.48	407.42 ± 77.86	<0.001
Image noise	25.73 ± 7.12	16.34 ± 6.12	<0.001
**SNR**			
AO	16.99 ± 5.16	28.34 ± 12.25	<0.001
LAD-P	26.43 ± 15.06	46.51 ± 37.70	<0.001
LAD-M	9.38 ± 5.74	12.48 ± 12.36	0.005
LAD-D	6.49 ± 5.20	7.80 ± 6.19	0.009
LCX-P	26.43 ± 15.06	46.51 ± 37.70	<0.001
LCX-M	13.19 ± 7.43	18.04 ± 12.48	<0.001
LCX-D	7.52 ± 4.95	9.74 ± 6.55	<0.001
RCA-P	26.42 ± 15.30	32.29 ± 30.99	<0.001
RCA-M	17.68 ± 11.92	25.75 ± 23.63	<0.001
RCA-D	13.43 ± 9.43	18.28 ± 12.75	<0.001
**CNR**			
AO	40.48 ± 32.74	80.65 ± 51.39	<0.001
LAD-P	42.01 ± 31.30	76.30 ± 48.96	<0.001
LAD-M	29.57 ± 23.41	52.60 ± 39.45	<0.001
LAD-D	23.96 ± 18.87	48.83 ± 33.68	<0.001
LCX-P	42.01 ± 31.30	76.30 ± 48.96	<0.001
LCX-M	32.59 ± 27.49	64.91 ± 43.19	<0.001
LCX-D	25.59 ± 22.75	51.92 ± 33.44	<0.001
RCA-P	42.71 ± 29.89	76.80 ± 50.84	<0.001
RCA-M	32.79 ± 27.09	68.40 ± 45.64	<0.001
RCA-D	34.06 ± 22.97	60.73 ± 39.33	<0.001
**SHARPNESS**			
MTF50	0.0091 (0.0046, 0.0137)	0.0093 (0.0051, 0.0164)	0.036
MTF50P	0.0088 (0.0046, 0.0128)	0.0090 (0.0059, 0.0136)	0.049
MTF30	0.0134 (0.0072, 0.0217)	0.0211 (0.0146, 0.0258) (0.231,0.001)	0.122

SE, smooth 3D+ combined with edge-2; CT, computed tomography; SNR, signal-to-noise ratio; CNR, contrast-to-noise ratio; HU, household; AO, lumen of the aortic root; LAD-P, M, D, left anterior descending artery, proximal, middle, and distal; LCX-P, M, D, left circumflex artery, proximal, middle, and distal; RCA-P, M, D, right coronary artery, proximal, middle, and distal; MTF, Modulation Transfer Function. Significance level, P < 0.05.

Due to individual differences in coronary artery anomalies, the image quality score was calculated based on three main coronary arteries (i.e., LAD, LCX, and RCA). The consistency of the two readers was good or perfect for each vessel (LAD, LCX, and RCA) and total vessels (kappa: 0.71–0.84), and the image quality scores in the SE group were significantly lower (i.e., better) than those in the routine group in each vessel and total vessels (all *P*s < 0.001). Image quality scores of three main coronary arteries (LAD, LCX, and RCA) and total segments improved to different degrees after removing image quality score 1 of those segments (percent: 49–70%) (Table 4).

**Table 4:** Comparison of image quality scores between the routine and SE groups.

		**LAD**			**LCX**			**RCA**			**Total**		
		**R1**	**R2**	**ka**.	**R1**	**R2**	**ka**.	**R1**	**R2**	**ka**.	**R1**	**R2**	**ka**.
Score (Median (25%,75%)	Routine group	3 (2, 3)	3 (2, 3)	0.81	3 (3, 4)	3 (3, 4)	0.83	3 (2, 3)	3 (2, 3)	0.84	3(2, 4)	3(2, 4)	0.83
	SE group	2 (1, 3)	2 (1, 3)	0.78	2 (2, 3)	3 (2, 3)	0.80	2 (1, 3)	2 (1, 2)	0.71	2(1, 3)	2(1, 3)	0.77
	*p*	<0.001	<0.001	<0.001	<0.001	<0.001	<0.001	<0.001	<0.001		<0.001	<0.001	
Improved Vessels (Number, %)		48(57)	59 (60)		51 (49)	60 (56)		40 (69)	57 (69)		139(70)	176(69)	

SE, smooth 3D+ combined with edge-2; LAD, left anterior descending artery; LCX, left circumflex artery; ka., Kappa; R, radiologist; RCA, right coronary artery.

### Diagnostic accuracy

Among the 90 patients, 35 underwent DSA for stenosis evaluations after CCTA imaging. Based on the results of DSA, the accuracy of the findings related to stenosis in these 35 patients was compared among the three groups (routine group, SE group, and DSA group). The results showed no statistically significant difference in the accuracy of the stenosis evaluations between the SE and routine groups at the per-vessel and total levels (all *P*s > 0.05). However, the SE group showed a slightly higher NPV on the LAD and RCA stenosis evaluations, as well as total NPV. The SE group also showed slightly higher sensitivity and accuracy than the routine group on the RCA stenosis evaluation. Moreover, eight segments were evaluated with CAD-RADS 6 (non-diagnostic situation) in the routine group, while two segments of RCA from these eight segments were improved to the diagnostic situation (Table 5).

**Table 5:** Comparison of diagnostic accuracy among the routine group, SE group, and the DSA group for coronary artery stenosis on per-segment and total levels.

	**LAD**		**LCX**		**RCA**		**total**	
	**Routine**	**SE**	**Routine**	**SE**	**Routine**	**SE**	**Routine**	**SE**
FPR, %	0.36	0.39	0.43	0.43	0.41	0.41	0.39	0.40
FNR, %	0.04	0.03	0.02	0.02	0.01	0.00	0.02	0.02
PPV, %	0.85	0.84	0.84	0.84	0.91	0.89	0.87	0.87
NPV, %	0.88	0.90	0.92	0.92	0.94	1.00	0.90	0.93
Se, %	0.96	0.97	0.98	0.98	0.99	1.00	0.98	0.98
Sp, %	0.64	0.61	0.57	0.57	0.59	0.59	0.61	0.60
Accuracy, %	0.86	0.86	0.86	0.86	0.91	0.92	0.88	0.88
*p* (routine vs. SE)	0.839		1.000		0.418		0.776	
Non-diagnostic segment	1	1	1	1	6	4	8	6

SE, smooth 3D+ combined with edge-2; FPR, False Positive Rate; FNR, False Negative Rate; PPV, Positive Predictive Value; NPV, Negative Predictive Value; Se, Sensitivity; Sp, Specificity; LAD, left anterior descending artery; LCX, left circumflex artery; RCA, right coronary artery; DSA, digital subtraction angiography.

## Discussion

**Figure 4:**
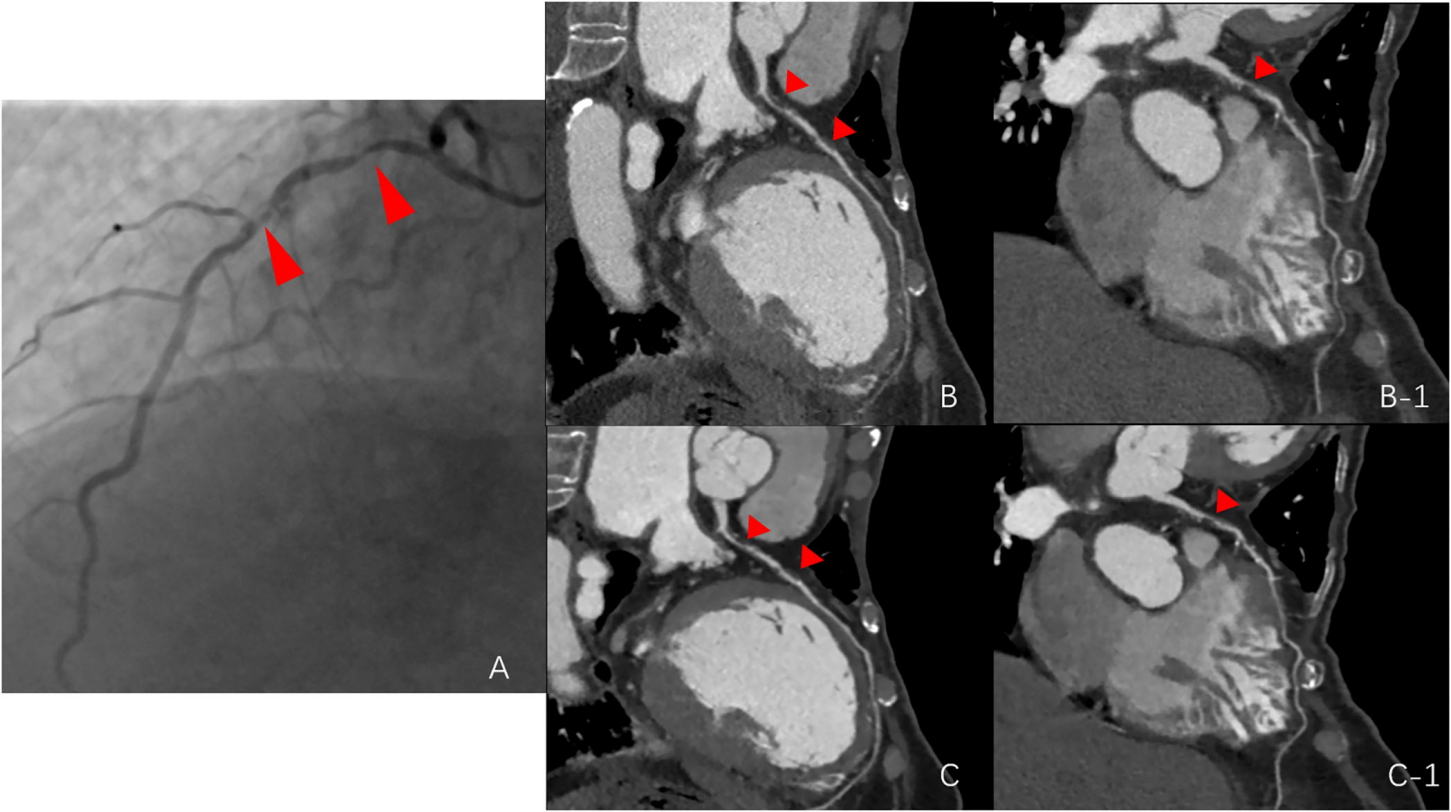
Comparison among the routine group, SE group, and digital subtraction angiography (DSA) group. **(A)** stenosis of the DSA group (red arrows point to the left anterior descending artery); **(B,B-1)**, stenosis of the routine group; and **(C,C-1)**, stenosis of the SE group.

Our study found that the application of a combination of image filters (smooth 3D+ and edge-2) on CCTA images significantly improved SNR and CNR values, due to ~36% image noise reduction with the use of the smooth 3D+ image filter. Importantly, we showed that image sharpness was significantly better in the SE group than in the routine group in terms of MTF50 and MTF50P. Notably, image sharpness is usually considered a desirable image quality for evaluating vessel lesions on CCTA (23, 30, 31) (Figure 4). Moreover, previous studies have also reported additional edge rise distance as a quantitative measure for image sharpness evaluation (7, 31). However, in our study, similar MTF30 between the groups indicated that the image sharpening ability of the SE group was limited.

Image quality scores were significantly lower in the SE group than in the routine group, indicating better image quality in the SE group. Consistent with the image quality scores assigned by the observers for the three main coronary arteries, the better quantitative image quality and image sharpness enhanced the diagnostic confidence of observers. In the group-wise comparison of diagnostic accuracy against the DSA results, no significant differences were observed between these two groups. However, in the routine group, 8 out of 105 coronary arteries had a CAD-RADS of 6, indicating poor image quality. Moreover, obstructive CAD could not be excluded, and 2 of the 8 arteries changed from undiagnosable to diagnosable, consistent with the DSA stenosis degree (<50%), indicating that the diagnostic accuracy in the SE group was improved compared to that in the routine group.

A major strength of our study is that our intervention could be implemented using readily available post-processing software on the workstation or console and will thus be easy to replicate because this technique is based on image domain processing instead of the raw data domain reconstruction. The image filter function on the AW4.7 workstation is convenient to use by any radiological worker whenever necessary. Moreover, this method improves image quality and increases radiologists' diagnostic confidence without increasing the cost or radiation dose to the patients. CCTA is a very mature imaging method in the evaluation of coronary stenosis and has relatively high diagnostic accuracy. Thus, improving the post-processing algorithms would not expect to dramatically improve the diagnostic accuracy, unless the original images do not meet the diagnostic requirements. In our study, the original CCTA images were from routine examinations and already had relatively high diagnostic accuracy. Even though the use of image filters provided improvement in some of the diagnostic parameters, such as NPV, the main effect of this image filter combination was the improvement of image quality and readers' diagnostic confidence. Nevertheless, our concept and results could be helpful for future CCTA applications. For example, the ability to reduce image noise with the combined filters could be used to further reduce the required radiation dose for patients. The improved diagnostic confidence may be beneficial in speeding up the workflow and relieving the pressure on radiologists who need to read a lot of images every day.

**Figure 5:**
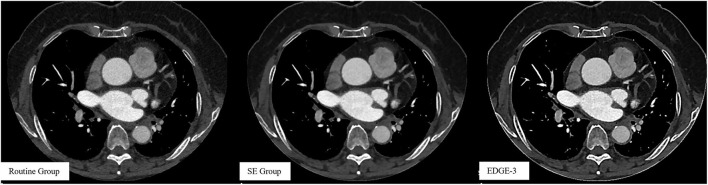
Images without using a combination of image filters in the routine group and images after using a combination of image filters in the SE (smooth 3D+ and edge-2) and Edge-3 (smooth 3D+ and edge-3) groups.

Our study has several limitations. First, the study participants were retrospectively recruited from a single institution, and the sample size was small. And only one-third of the patients had undergone DSA due to the fact that if CCTA showed negative results, patients were less likely to undergo DSA. However, this intra-individual study with such a sample size clearly showed significant improvements in quantitative measurements, including image noise and sharpness and qualitative image evaluation. Second, we only used the combination of smooth 3D+ and edge-2 in the research group but did not compare this combination and other combinations. Smooth 3D+ was chosen as the highest level of reducing image noise and edge-2 was only the second highest level of edge sharpening because the highest level (edge-3) showed excessive sharpening with a severe “dark ring” around the edge (32, 33) (Figure 5). Therefore, the combination of image filters (smooth 3D+ and edge-2) was investigated in our study, and our results demonstrated that this combination balanced the image noise and spatial resolution and remarkably improved image quality. Third, the effect of the image filter was evaluated on CCTA images obtained by only GE CT. It remains unknown whether similar results will be observed if CT scanners from other vendors are used. However, conceptually our results clearly supported our hypothesis.

In conclusion, this study showed that the use of an additional image filter combining smooth 3D+ and edge-2 in the image domain on an AW could improve the image quality of CCTA and increase radiologists' diagnostic confidence.

## Summary

Smoothing and edge-enhancement filters may be combined to improve the image quality and diagnostic confidence in CCTA. Image domain filters such as smooth 3D+ and edge-2 are readily available on an AW and are easy to be applied.

## Data availability statement

The raw data supporting the conclusions of this article will be made available by the authors, without undue reservation.

## Ethics statement

The studies involving human participants were reviewed and approved by Ethics Committee of Huadong Hospital. Written informed consent for participation was not required for this study in accordance with the national legislation and the institutional requirements.

## Author contributions

LJ and ML: conception, design, and study supervision. LJ and PG: development of methodology. LJ, PG, KW, and ML: acquisition of data (provided animals, acquired and managed patients, and provided facilities, etc.). LJ, JL, and ML: analysis and interpretation of data (e.g., statistical analysis and computational analysis). LJ, PG, and ML: writing, review, and/or revision of the manuscript. All authors contributed to the article and approved the submitted version.

## Funding

This work was supported by the Youth Medical Talents—Medical Imaging Practitioner Program [Grant number, AB83030002019004], the Health Commission of Shanghai [Grant number, 2018ZHYL0103], the National Natural Science Foundation of China [Grant number, 61976238], and Fudan University.

## Conflict of interest

Author JL was employed by GE Healthcare China. The remaining authors declare that the research was conducted in the absence of any commercial or financial relationships that could be construed as a potential conflict of interest.

## Publisher's note

All claims expressed in this article are solely those of the authors and do not necessarily represent those of their affiliated organizations, or those of the publisher, the editors and the reviewers. Any product that may be evaluated in this article, or claim that may be made by its manufacturer, is not guaranteed or endorsed by the publisher.

## Abbreviations

AO: aortic root
CAD: coronary artery disease
CAD-RADS: Coronary Artery Disease - Reporting and Data System
CCTA: Coronary computed tomography angiography
CNR: contrast-to-noise ratio
DICOM: Digital Imaging and Communications in Medicine
DLP: dose-length product
DSA: digital subtraction angiography
IRs: iterative reconstruction algorithms
LAD-D: distal left anterior descending artery
LAD-M: middle left anterior descending artery
LAD-P: proximal left anterior descending artery
LCX-D: distal left circumflex artery
LCX-M: middle left circumflex artery
LCX-P: proximal left circumflex artery
MTF: modulation transfer function
NPV: negative predictive value
PVAT: perivascular adipose tissue
RCA-D: distal-proximal right coronary artery
RCA-M: middle right coronary artery
RCA-P: proximal right coronary artery
SNR: signal-to-noise ratio
SSF: Snapshot Freeze
SD: standard deviation
RKs: reconstruction kernels
ROI: region of interest
WC/WW: window center-window width.
